# Plasma parathyroid hormone response to vitamin D3 supplementation among women of reproductive age: A randomized double-blind placebo-control trial

**DOI:** 10.1371/journal.pone.0276506

**Published:** 2022-11-10

**Authors:** Megan Chong Hueh Zan, Melissa Leong En Ying, Loke Seng Cheong, Khor Geok Lin

**Affiliations:** 1 Division of Nutrition and Dietetics, International Medical University, Kuala Lumpur, Malaysia; 2 Faculty of Medicine and Health Sciences, University of Auckland, Auckland, New Zealand; Oregon State University, UNITED STATES

## Abstract

While vitamin D inadequacy occurs worldwide, there is a lack of consensus internationally on the optimum plasma levels of 25(OH)D to maximally suppress the level of parathyroid hormone toward reducing bone loss. This study aimed to investigate the response of intact parathyroid hormone (iPTH) to vitamin D3 supplementation among Malaysian women of reproductive age in a randomised double-blind placebo-control trial [NMRR-15-479-25680]. A total of 106 women who fulfilled the study inclusion criteria were randomly assigned to receive daily one of these three supplement doses (i) 600 IU vitamin D3 + 500 mg calcium; (ii) 1200 IU vitamin D3 + 500 mg calcium; or (iii) 4000 IU vitamin D3 + 500 mg calcium. The placebo group received daily 500 mg calcium. The outcome examined was change in plasma iPTH concentration in response to daily vitamin D3 supplementation for 16 weeks. Fasting blood sample was obtained at baseline and post-supplementation. A total of 78 subjects (73.6%) completed the intervention. None of the supplementation groups brought about any detectable suppression of iPTH concentration post-supplementation. Vitamin D3 supplementation resulted in overall increase in plasma 25(OH)D levels, but only the 4000 IU/day group showed a significant dose effect post-supplementation (mean 49.7 ± 26.5 nmol/L) compared to placebo (29.3 ± 13.3 nmol/L). The lack of iPTH suppression is attributed to high prevalence of vitamin D insufficiency at baseline and the supplementation regimen was inadequate to raise the 25(OH)D level to cause PTH suppression. Inadequate calcium intake of the participants was also a likely contributing factor to the result. As prolonged vitamin D insufficiency and hypocalcaemia could lead to a compensatory rise in PTH resulting in accelerated bone loss, as well as posing increasing risks of non-skeletal morbidities, further clinical trials with an adequately powered sample size should be undertaken over an appropriate study duration to verify the results obtained in this study.

## Introduction

Globally, vitamin D deficiency has been reported in all age groups [1–5] (. Exclusively breastfed infants, dark-skinned individuals, older adults, those living in northern latitudes and people with habitually limited sun exposure owing to clothing cover or use of sunblock are at particularly high risk for vitamin D deficiency. Vitamin D deficiency is characterized by impaired bone mineralization manifesting as the childhood bone disease of rickets or osteomalacia in adults.

Circulating concentrations of serum or plasma vitamin D as 25(OH)D, also called calcidiol, has long been recognized as an indicator of vitamin D status primarily in relation to skeletal health outcomes [6, 7]. In its biologically active form as1,25-dihydroxycholecalciferol, [1,25(OH)2D] also called calcitriol, vitamin D functions to maintain calcium and phosphate homeostasis by regulating their intestinal absorption and deposition in bone. The renal activation of 25(OH)D to 1,25(OH)2D is regulated by parathyroid hormone (PTH) in response to low intake or absorption of calcium and/or low vitamin D status. The compensatory increase in the production of PTH has a twofold effect: (i) triggering increased renal hydroxylation of 25(OH)D to 1,25(OH)2D, thus making more of the active metabolite available; and (ii) increasing bone resorption, thereby mobilizing calcium from the bones to maintain serum calcium concentration in the physiological range [8]. PTH level is thus elevated in vitamin D insufficiency, but when prolonged, can lead to progressive bone loss. Nonetheless, the plasma levels of 25(OH)D sufficient to keep the PTH level at a range that will prevent bone loss remain unclear.

The concentration or threshold of 25(OH)D needed to maximally suppress serum PTH has been suggested as a measure of optimal vitamin D status [9]. However, there is a lack of international consensus on what constitutes an optimum 25(OH)D concentration. Values between 30 and 80 nmol/L have been suggested based on findings from various studies, largely in postmenopausal and elderly women [9, 10]. In the international Multiple Outcomes of Raloxifene Evaluation study, a large prospective intervention trial in postmenopausal women with osteoporosis, Lips et al. [11] reported a significant negative correlation between serum 25(OH)D and serum PTH in all geographical regions. A study of nearly 17,646 25(OH)D and 5,579 PTH samples from men and women with a mean age of 62 years from Southeast China, reported the optimal serum threshold of 25(OH)D for bone health was between 40 and 50 nmol/L [12]. A similar range of serum 25(OH)D concentration of 40–50 nmol/L was shown to prevent a rise in PTH concentration among calcium-sufficient African American women aged 50–75 years [10]. Meanwhile, Olmos et al. [13] suggested a threshold of serum 25(OH)D level of 75 nmol/L as necessary for the prevention of secondary hyperparathyroidism and hip bone loss for a Spanish community comprising mostly postmenopausal women.

There are relatively fewer reports on the relationship between vitamin D threshold and PTH concentration in premenopausal women, despite high prevalence of hypovitaminosis D in this population group, including among Asian women [14–17]. Against this background, a randomised double-blind placebo-control trial was conducted to investigate the response of plasma intact PTH to vitamin D3 supplementation among women of reproductive age from urban locations in Malaysia. The primary outcome examined was the change in plasma iPTH concentration in response to daily supplementation of 600 IU, 1200 IU, or 4000 IU oral vitamin D3 for 16 weeks.

## Methods

### Ethical considerations

Ethical approval to conduct the study was granted by the International Medical University Joint Research and Ethics Committee (IMU-JC)–project ID: IMU R 144/2014 –File II in May 2015. This study was registered prospectively with the Medical Research Ethics Committee, Ministry of Health, Malaysia [NMRR-15-479-25680] in May 2015 before enrolment of subjects. The NMRR is the national trials registry of Malaysia. This trial was also registered retrospectively under ClinicalTrials.gov, [NCT05281107] on 15 March 2022.

### Study design

This study was designed as a randomised double-blind placebo-control trial to investigate the response of intact parathyroid hormone (iPTH) to vitamin D3 supplementation. A computer-generated allocation list of random permuted blocks by researcher was used to ensure concealment of allocation to subjects and investigators. The participants received individual specific code and were randomised by 1:1:1:1 allocation ratio into the three supplementation and control groups. The code-break were performed upon completion of the study analysis. Code breaking was also permitted in the event of a serious adverse event.

### Sample size, subjects and supplements

Calculation of sample size was done using the statistical power analysis program, G*Power 3. Considering the study design with four groups and two measurements (difference before and after supplementation), the repeated measures ANOVA between and within groups’ interactions were suggested as the statistical test in G*Power software. A partial eta square of 0.06, corresponding to a medium Cohen’s effect size was applied. This gives an effect size F of about 0.25, and with that, an alpha significance of 0.05 and a power set at 0.99, the minimum generated sample size for the four groups was 100 participants (25 participants per arm). Taking into consideration that this supplementation study faces the risk of dropouts, the minimum sample size was increased by 20% to a total of 120 participants.

Women aged 20–45 years were recruited by using convenience sampling from among the staff and students in two universities located in Kuala Lumpur and Selangor state between July 2015 and April 2016. Recruitment was carried out through direct contacts, emails and flyers. Women were excluded if they were pregnant, breastfeeding, having chronic illnesses or taking medications that might affect vitamin D metabolism (e.g., thiazide diuretics, prednisolone).

**Fig 1:**
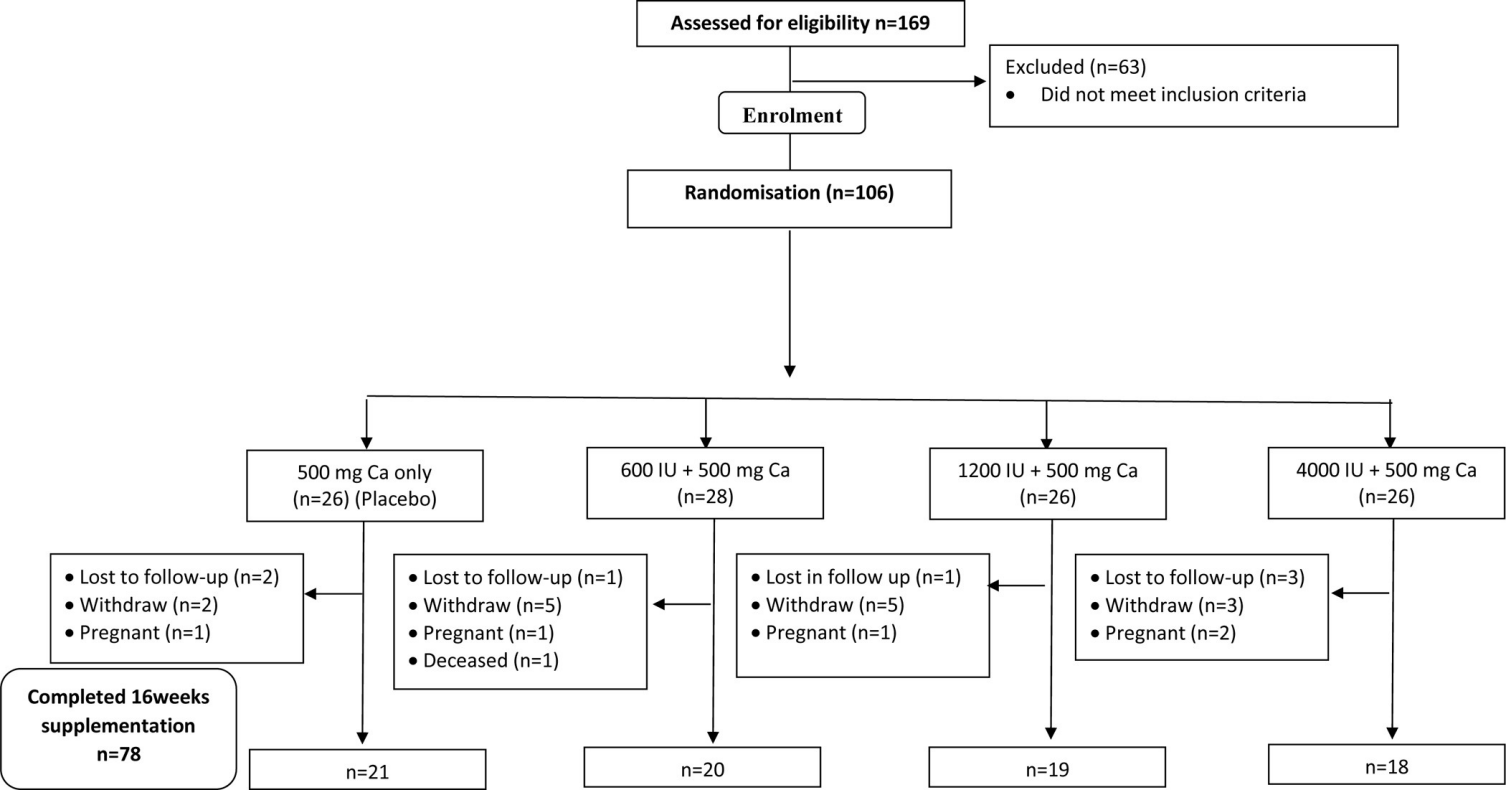
Consolidated standards of reporting trials flow diagram for enrolment in clinical trial of vitamin D supplementation.

A total of 106 women who fulfilled the inclusion criteria of the intervention study were randomly assigned to receive one of these three supplement doses (i) 600 IU vitamin D3+ 500 mg calcium; (ii) 1200 IU vitamin D3 + 500 calcium; or (iii) 4000 IU vitamin D3 + 500 calcium. The placebo group was allotted daily 500 mg calcium (Fig 1). Out of the three supplementation doses, the smallest amount of 600 IU/day (equivalent to 15 μg/day) was based on Malaysia’s recommended daily intake of 15 μg/day of vitamin D for women aged 19–65 years [18], while the highest supplementation dose of 4000 IU/day (equivalent to 100 μg/day) was guided by the tolerable upper intake level of 100 μg/day for 18 years and older adults suggested by IOM [19]. As for the magnitude of the calcium dose provided, this was based on previous studies that the mean dietary calcium intake of Malaysian women was reported to be 400–500 mg /day [20–22]. Hence, in order to meet approximately the daily dietary intake recommendation of calcium for Malaysian women of reproductive age (1000 mg) [18], every participant was allotted daily 500 mg calcium. In regard to the choice of 16 weeks for the intervention, the duration was based on the general guidance that vitamin D supplementation takes 4–5 time the 3-weeks half-life to reach a steady state and re-measurements of serum 25(OH)D are recommended not be done earlier than 8 weeks after starting with a daily vitamin D supplement [23].

The vitamin D3 and calcium supplements were packed into powder sachets to be taken with 150 ml room temperature water. The vitamin D3 concentration for each study group was tested by an independent laboratory using high-performance liquid chromatography (HPLC) method and verified to meet at least 90% of the intended dosage. The intervention supplements were similar in taste, appearance and odour for all groups. The supplements were distributed to the participants on a weekly basis. Close monitoring and follow-up with the participants were carried out bi-weekly by sending reminders through text messages and phone calls. Compliance was monitored by calculating any remaining sachets brought back by the participants prior to giving them the following supply of supplements. As for checking for safety and tolerance in taking the supplements, the participants were interviewed every week for any experience of gastrointestinal discomfort (e.g., bloating, nausea, diarrhoea, vomiting).

### Data collection and measurements

Data collection including measurements began after the participants had given their written consent. As the vitamin D status of individuals is recognised to reflect the sum effect of various factors, data collected in this study included socio-demographic background using a structured questionnaire, dietary intake based on 24-hour recall method for two days, anthropometric measurements using standard procedures, and self-reported sunlight exposure based on a questionnaire validated for Malaysian individuals.

Body weight and height were measured using respectively a digital scale (TANITA HD-314, Tanita Corporation, Tokyo, Japan)) and a microtoise measuring tape (SECA 206 Mechanical Measuring Tape, SECA Group, Hamburg, Germany) following the International Society for the Advancement of Kinanthropometry (ISAK) procedures, with two measurements being recorded to obtain the mean measurement. Body mass index (BMI) status was computed based on the WHO BMI [24] classification cut-off point. Waist circumference, a surrogate indicator for central obesity was measured using a measuring tape at the level of the narrowest part of the waist between the lower costal (10^th^ rib) border and the top of the iliac crest, perpendicular to the long axis of the trunk. Two measurements were recorded to obtain a mean reading of the waist circumference.

Participants were instructed to self-record their dietary intake using a 24-hour recall method over two days, including one weekday and one weekend. Dietary intake data were collected at baseline and at the end of the intervention. The intake data were analysed using Nutritionist Pro Diet Analysis Software (NutriPro Software) based on Nutrient Composition of Malaysian Foods [25], Singapore Nutrient Composition of Foods [26] and product nutrient labels. In addition, vitamin D intake was assessed separately using a semi-quantitative food frequency questionnaire (FFQ) adapted from Zaleha et al. [27], followed by analysis based on information from product labels, and the food composition tables of Malaysia, Singapore and Thailand.

At baseline, self-reported sunlight exposure was determined using a questionnaire validated for Malaysian subjects [28]. Participants were asked to record their outdoor activities during the previous one week, in terms of type of activity, usual time of activity exposed to sunlight, duration (in hours), frequency (per week), usual outdoor attire and use of sunscreen and umbrella when outdoor.

Fasting blood samples were drawn by a physician or phlebotomist. Blood samples (5 ml) were taken by venepuncture following overnight fast, once at baseline and at the end of intervention. Plasma samples were extracted by centrifuging the whole blood at 4000 rpm for 10 minutes, and stored at -80˚C until analysed. Vitamin D status was assessed by determining plasma 25(OH)D concentration given its stability and longer half-life than the activated 1,25(OH)D metabolite. Plasma 25(OH)D and the biologically active intact parathyroid hormone (iPTH) were determined using the ADVIA Centaur XP Immunoassay System (SIEMENS, Ireland).). The Centaur Vitamin D Total Assay employed a proprietary monoclonal with minimal 1.1% 3-epi-25(OH)D cross-reactivity aligned to the LC-MS/MS 25(OH)D Reference Measurement Procedure (RMP), the reference procedure for the Vitamin D Standardisation Program (VDSP). The assay has a broad dynamic range (4.2–150 ng/mL) and good precision (4.2%-11.9% coefficient of variation) for vitamin D measurements, and 4.6–2200 pg/mL (0.488–233 pmol/L) for intact PTH measurement. The classification of vitamin D status in relation to plasma 25(OH)D levels was based on the guidelines of the Institute of Medicine [29].

### Data analysis

Statistical analysis was conducted using the statistical package for social sciences (SPSS) version 21 software (SPSS Inc., Chicago, IL, U.S.). ANOVA test was conducted to determine for significant differences between the mean plasma 25(OH)D and intact PTH concentrations among the groups. The alpha significance level was set at 0.05, while bivariate testing was done using Tukey’s HSD which is adjusted for multiple comparisons. The results of the randomized control trial were analysed by the Intention To Treat (ITT) statistical concept, which is based on the principle that all participants who have been randomly assigned should be analysed, regardless of the actual treatment received, and regardless of subsequent withdrawal from treatment [30]. The ITT analysis is recognized as a potential solution to address the common dual problems faced by RCTs namely, noncompliance and missing outcomes [31]. (Gupta, 2011) [30]. It is said that applying the ITT principles yields an unbiased estimate of the efficacy of the intervention on the primary study outcome at the level of adherence observed in the trial [32], and ITT is often regarded as least biased [33].

## Results

### Supplementation compliance

Out of 106 participants who were randomized into the four arms of the supplementation intervention, a total of 78 (73.6%) completed the 16 weeks study. The supplements were generally well tolerated by the participants. Out of the 28 participants who did not complete, the majority (n = 15) withdrew for reasons including being unable to tolerate the side effects in taking the supplements (bloating and nausea), and unable to comply with the consumption regime. The other reasons included loss to follow-up (n = 7), pregnancy (n = 5) and death (n = 1) due to reasons unrelated to the study.

No other side effects (e.g. diarrhoea, vomiting) or adverse events were reported throughout the study. A post-hoc test was conducted determining the power of samples recruited in this study. A standard effect size of 0.5, two-tailed, alpha significance of 0.05, and the total sample size of 78 that completed the study were input into the G*Power test. Power obtained from a total sample of 78 participants was 0.99. The results at the end of the supplementation were analysed and reported below for a total of 106 participants, based on the Intention to Treat (ITT) basis, as explained in the Methods section.

### Background characteristics of participants at baseline

The participants comprised the main ethnicity groups of Malaysia namely, Malay (n = 46, 43.4%), Chinese (n = 43, 40.6%) and Indian (n = 17, 16%) (Table 1). They had a mean age of 26.1 ± 5.8 years (mean±SD), and the majority possessed tertiary education. While two-thirds (66%) had normal body mass index (BMI = 18.5–24.9 kg/m^2^), 17.9% were overweight (BMI = 25.0–29.9 kg/m^2^), and another 9.4% were obese (BMI ≥ 30.0 kg/m^2^). Nonetheless, the majority of them (79.2%) did not manifest central obesity (waist circumference ≥ 80 cm). The participants self-estimated sun exposure at median of3.9 (1.9–8.1) hours /week. At baseline, there were no significant differences among the placebo and supplementation groups with respect to their socio-demographic characteristics, BMI, central obesity status, and self-reported weekly sun exposure duration.

**Table 1:** Baseline characteristics of participants compared by supplementation dose (N = 106).

	Placebo 500mg Ca	600 IU vitD_3_ + 500 mg Ca	1200 IU vitD_3_ + 500 mg Ca	4000 IU vitD_3_ + 500 mg Ca	All participants	p-value
**Participants (n)**	26	28	26	26	106	
**Age (years)^†^**	25.7 ± 5.6	25.8 ± 5.4	26.8 ± 6.8	26.0 ± 5.8	26.1 ± 5.8	0.906
**Ethnicity^¥^**						
Malay	11 (42.3)	13 (46.4)	12 (46.2)	10 (38.5)	46 (43.4)	
Chinese	10 (38.5)	10 (35.7)	11 (42.3)	12 (46.2)	43 (40.6)	
Indian	5 (19.2)	5 (17.9)	3 (11.5)	4 (15.4)	17 (16.0)	
**Marital Status^¥^**						
Single	23 (88.5)	22 (78.6)	22 (84.6)	23 (88.5)	90 (85.0)	
Married	3 (11.5)	6 (21.4)	4 (15.4)	3 (11.5)	16 (15.0)	
**Education Level^¥^**						
Secondary school	1 (3.8.)	0 (0.0)	2 (7.7)	1 (3.8)	4 (3.8)	
Pre-university	5 (19.2)	4 (14.3)	6 (23.1)	6 (23.1)	21 (19.8)	
University	20 (76.9)	24 (85.7)	18 (69.2)	19 (73.1)	81 (76.4)	
* **Household income/month**						
<RM 5000	25 (96.2)	27 (96.4)	25 (96.2)	25 (96.2)	102 (96.2)	
>RM 5000	1 (3.8.)	1 (3.6)	1 (3.8)	1 (3.8)	4 (3.8)	
**Weight (kg)^§^**	56.9 (49.7–61.9)	56.6 (51.6–66.8)	57.5 (47.4–65.3)	55.1 (50.1–61.2)	56.0 (50.2–63.9)	0.977
**Height (cm)^†^**	158.6 ± 5.5	157.2 ± 5.7	158.2 ± 5.7	157.8 ± 3.9	157.9 ± 5.2	0.801
** **BMI (kg/m^2^)^§^**	22.0 (19.8–25.4)	22.4 (19.9–26.1)	21.9 (20.3–26.0)	21.8 (20.3–25.6)	22.0 (20.3–25.5)	0.934
Underweight (<18.5)	3 (11.5)	1 (3.6)	2 (7.7)	1 (3.8)	7 (6.6)	
Normal (18.5–24.9)	15 (57.7)	20 (71.4)	17 (65.4)	18 (69.2)	70 (66.0)	
Overweight (25.0–29.9)	7 (26.9)	4 (14.3)	3 (11.5)	5 (19.2)	19 (17.9)	
Obese (≥30.0)	1 (3.8)	3 (10.7)	4 (15.40	2 (7.7)	10 (9.4)	
**^#^Waist circumference (cm)^§^**	72.6 (66.3–78.4)	71.6 (65.6–75.9)	71.6 (66.5–79.5)	69.9 (66.8–79.2)	71.3 (66.5–77.9)	0.972
Normal (<80)	21 (80.8)	23 (82.1)	20 (76.9)	20 (76.9)	84 (79.2)	
Central obesity (≥80)	5 (19.2)	5 (17.9)	6 (23.1)	6 (23.1)	22 (20.8)	
**Sun exposure** (hours per week)^§^	3.0 (1.8–7.7)	3.9 (1.5–5.9)	3.2 (1.8–8.1)	6.4 (2.4–9.8)	3.9 (1.9–8.1)	0.621
**Dietary vitamin D intake**						
IU/day^§^	241 (164–359)	220 (173–291)	238 (156–436)	267 (182–450)	236 (167–354)	0.386
mcg/day^§^	6.03 (4.1–9.0)	5.5 (4.3–7.3)	6.0 (3.9–10.1)	6.7 (4.6–11.3)	5.9 (4.2–8.9)	
**Dietary calcium intake** (mg/day)	367±212	392±160	396±175	415±226	393 ± 192	0.852
**^##^Vitamin D status**(nmol/L)^¥^						
Deficiency (<30)	14 (53.8)	18 (64.3)	11 (42.3)	12 (46.2)	55 (51.9)	
Insufficiency (30–49)	10 (38.5)	10 (35.7)	10 (38.5)	11 (42.3)	41 (38.7)	
Sufficiency (≥50)	2 (7.7)	0 (0.0)	5 (19.2)	3 (11.5)	10 (9.4)	
**Plasma 25(OH)D** (nmol/L)^†^	27.7 ± 13.3	24.9 ± 11.5	34.5 ± 17.1	33.0 ± 12.3	29.9 ± 14.0	0.871
**Plasma Intact PTH (pmol/L)^†^**	3.8 ± 1.6	5.3 ± 2.9	4.6 ± 2.0	4.2 ± 1.4	4.5 ± 2.1	0.251

All significant values (p<0.05) were in boldface.

^§^ Skewed data were expressed as median (Q1-Q3), and compared by Kruskal-Wallis test at 0.05 significant values.

^**†**^ Continuous data were expressed as mean ± SD, and compared by one-way ANOVA at 0.05 significant values.

^¥^ Categorical data were expressed as n(%).

*RM5000 ~ USD1200 based on the exchange rate of 1 USD = 4.16 Malaysian Ringgit (3 Jan 2022)

**Body mass index (WHO, 1998).

^#^ (WHO, 2008).

^##^ (IOM, 2010).

^###^Reference range for plasma intact PTH as 1.95–8.49 pmol/L (ADVIA Centaur® Intact PTH Assay Specifications. SIEMENS)

Based on 24-hour dietary recall records for 2 days, the intake of vitamin D among the participants was markedly inadequate averaging 5.9 (4.2–8.9) μg/day, which amounts to scarcely one-third the daily dietary intake recommendation for vitamin D (15μg) of Malaysia. The main food sources of vitamin D were bread, malted milk beverage and condensed milk, which are commercially fortified with vitamin D. Other salient dietary sources of vitamin D included chicken, eggs, pork and anchovy. As for dietary intake of calcium, the overall mean daily intake was 393±192mg, derived mainly from fortified malted drinks and anchovy. There were no significant differences in dietary vitamin D and calcium intake among the study groups prior to the commencement of supplementation. None of the participants reported taking dietary supplements.

At baseline, the overall mean plasma 25(OH)D concentration of the participants stood at 29.9 ± 14.0 nmol/L (mean±SD), there being no significant differences amongst the supplementation and placebo groups. More than half of the participants (51.9%) showed 25(OH)D deficiency(<30nmol/L), while another 38.7% had2 5(OH)D levels between 30-49nmol/L. Thus, the majority of the participants (90.6%) showed 25(OH)D insufficiency (< 50 nmol/L).

The mean iPTH concentration of the participants before supplementation showed as 4.5±2.1 pmol/L (mean±SD), which appears to be within the reference range reported in other studies, including the range between 0.88–6.99 pmol/L cited for a large Chinese population [34], and between 1.95–8.49 pmol/L for laboratory assay specifications (ADVIA Centaur® Intact PTH Assay Specifications. SIEMENS). There were no significant differences in the iPTH concentrations at baseline among the study groups (p = 0.251).

### Primary study outcome: Change in plasma iPTH concentration in response to vitamin D3 supplementation for 16 weeks

**Fig 2:**
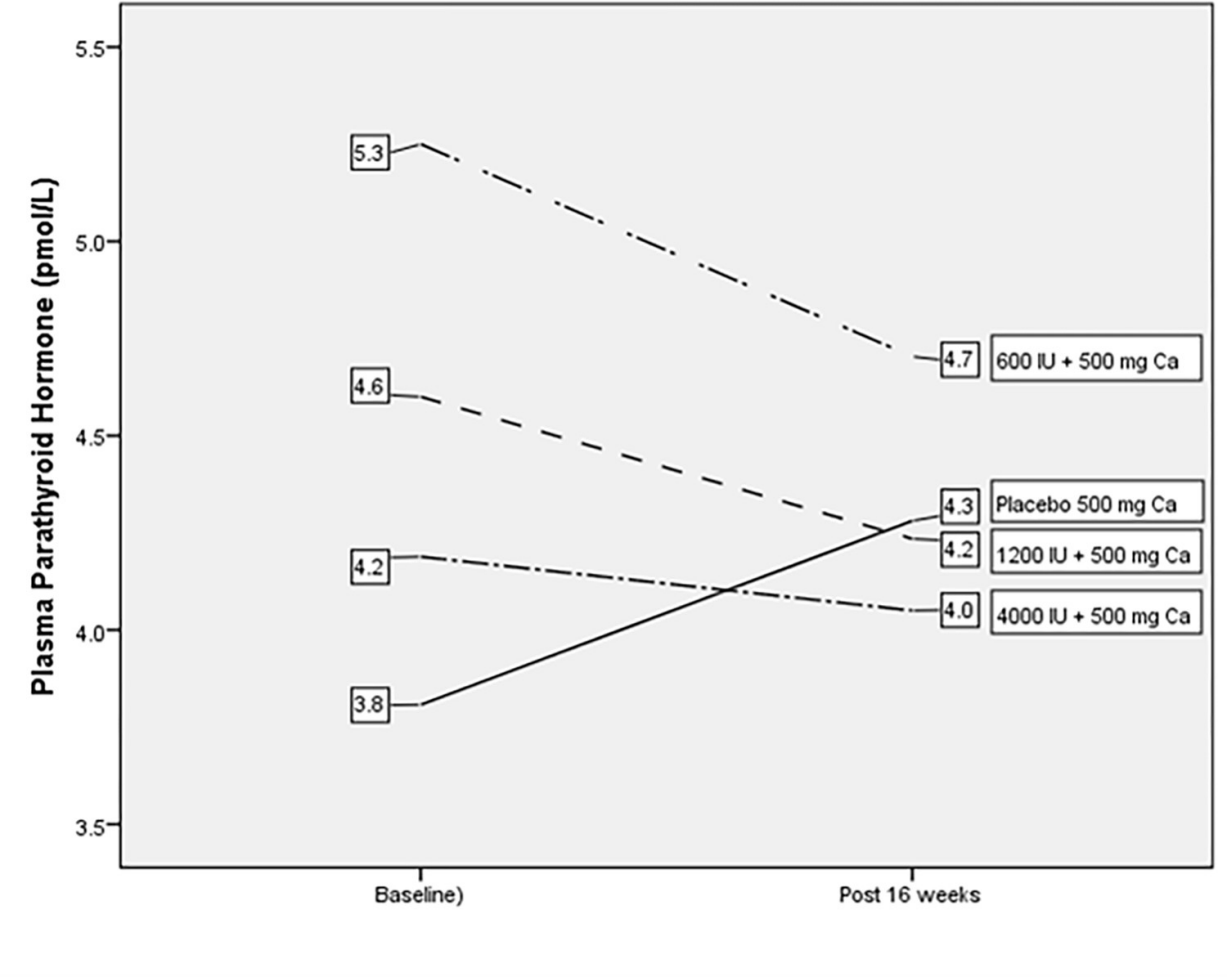
Plasma parathyroid hormone (pmol/L) before and after supplementation within groups for intention to treat analysis (N = 106).

At the end of 16 weeks, the 4000 IU/day, 1200 IU/day and 600 IU/day supplementation groups generated insignificantly small reductions in plasma iPTH, in the following manner: minus 0.1 ± 1.7%, minus 0.4 ± 1.7% and minus 0.5 ± 1.7%, respectively (Table 2). The placebo group recorded a change of plus 0.5 ± 2.7% (Table 2). In brief, vitamin D_3_ supplementation based on the magnitudes given in this study for 16 weeks did not result in any significant (p = 0.986) suppression of plasma iPTH level among the participants (Fig 2).

**Table 2:** Changes in plasma25-OHD and iPTH concentrations following vitamin D_3_ supplementation for 16 weeks (N = 106).

	Placebo 500mgCa n = 26	600 IU + 500 mg Ca n = 28	1200 IU + 500 mg Ca n = 26	4000 IU + 500 mg Ca n = 26	All participants n = 106	Adjusted ANCOVA comparison: 0 vs 600IU, p	Adjusted ANCOVA comparison: 0 vs 1200IU, p	Adjusted ANCOVA comparison: 0 vs 4000IU, p
^##^Vitamin D Status^¥^								
Deficiency (<30 nmol/L)	16 (61.5)	13 (46.4)	8 (30.8)	6 (23.1)	43 (40.6)			
Insufficiency (30–49 nmol/L)	6 (23.1)	11 (39.3)	8 (30.8)	9 (34.6)	34 (32.1)			
Sufficiency (≥50 nmol/L)	4 (15.4)	4 (14.3)	10 (38.5)	11 (42.3)	29 (27.4)			
25(OH)D(nmol/L)^†^	29.3 ± 13.3	32.9 ± 15.2*	41.7 ± 16.3*	49.7 ± 26.5*	38.3 ± 19.9	0.886	0.079	**0.001**
Change (from baseline)	1.6 ± 9.8	8.0 ± 14.1	7.2 ± 13.6	16.7 ± 23.5	8.4 ± 16.7			
% Change	5.8	32	21	51	27.4			
Serum Intact PTH (pmol/L)^†^	4.3 ± 2.1	4.7 ± 2.9	4.2 ± 2.5	4.0 ± 2.0	4.3 ± 2.4	0.920	1.000	0.986
Change (from baseline)	0.5 ± 2.7	-0.5 ± 1.7	-0.4 ± 1.7	-0.1 ± 1.7	-0.2 ± 2.0			

^¥^ Categorical data were expressed as n (%)

^**†**^ Continuous data were expressed as mean ± SD

Bivariate testing was done using Tukey’s HSD, and all significant values (p<0.05) were in boldface

*All significant values (p<0.05) from baseline.

## Based on IOM vitamin D level classification, 2010.

**Fig 3:**
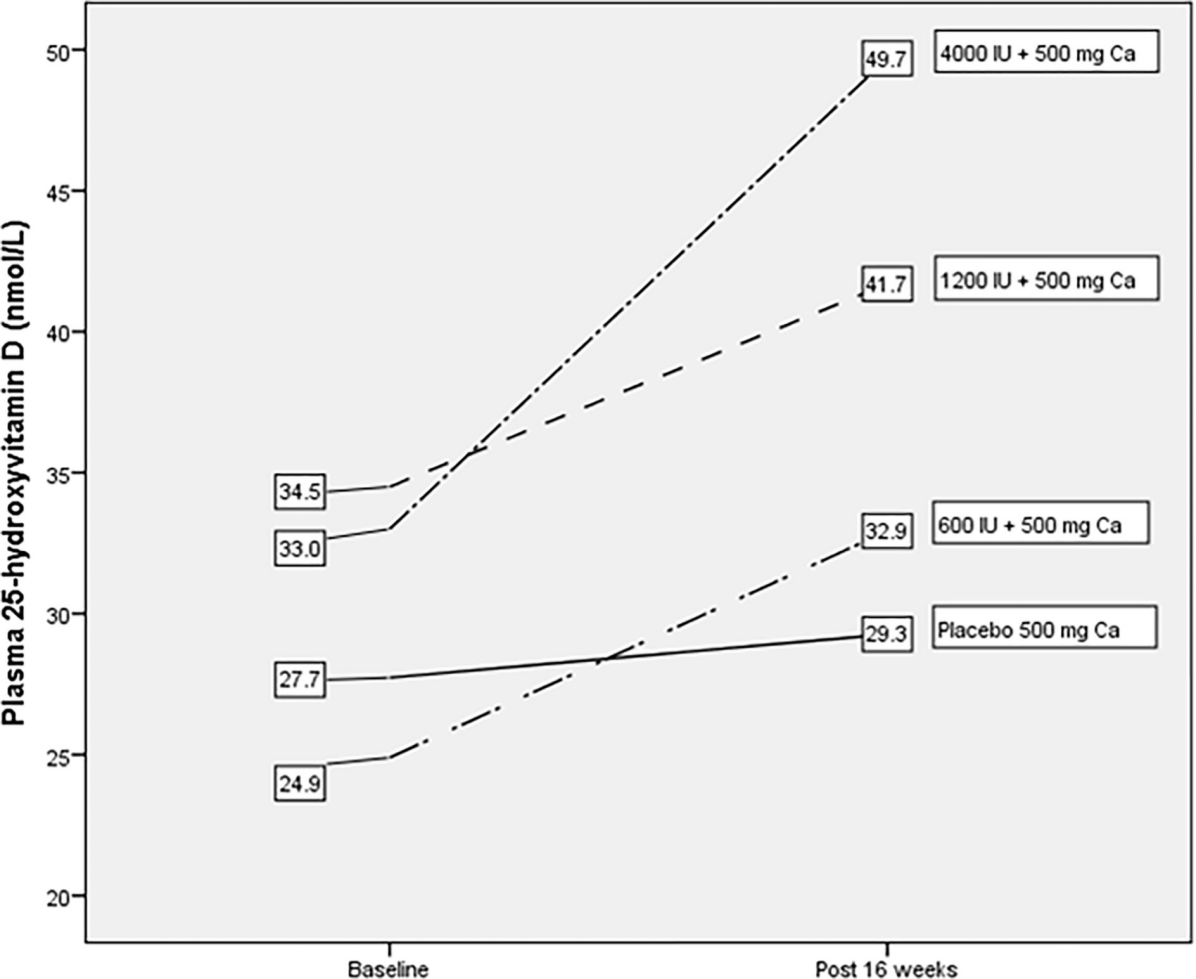
Plasma 25-hydroxyvitamin D (nmol/L) before and after supplementation within groups for intention to treat analysis (N = 106).

Vitamin D3 supplementation in this study showed an increased in plasma 25(OH)D concentration at the end of 16 weeks. The overall mean plasma 25(OH)D concentration stood at 38.3 ± 19.9 nmol/L (mean±SD) which denotes an increase of 27.4% from the baseline mean value of 29.9 ± 14.0 nmol/L (mean±SD) (Table 2). The individual supplementation groups of 4000 IU/day, 1200 IU/day and 600 IU/day showed increases in mean plasma 25(OH)D concentrations, constituting 51%, 21% and 32% respectively, almost in a dose response manner. The placebo group showed the lowest increase of 5.4%. Fig 3 illustrates the respective increases in plasma 25(OH)D levels brought about by the vitamin D3 supplementation from baseline to post-supplementation. Statistically however, only the 4000 IU/day group showed a significant (p = 0.001) increase when compared with the placebo group.

At the end of the 16 weeks intervention, 27.4% of the participants showed 25(OH)D sufficiency (> 50 nmol/L) compared to 9.4% at baseline, indicating an increase of 18% for 25(OH)D sufficiency (27.4% vs 9.4%) (Table 2). Each of the supplementation groups also showed individual increase in the prevalence of 25(OH)D sufficiency as follows: 30.8% increase (42.3% vs 11.5%) for the 4000 IU/day group; 19.3% increase (38.5% vs 19.2%) for the 1200 IU/day; and 14.3% increase (14.3% vs 0%) for the 600 IU/day. The placebo group showed the smallest increase of 7.7% (15.4% vs 7.7%). Overall, the prevalence of plasma 25(OH)D insufficiency (< 50 nmol/L) among the subjects persists at a relatively high level of 72.7%.

No significant changes were recorded for the study groups with regard to dietary intake, body weight status and sun exposure between baseline and post-supplementation (Table 3). Overall, vitamin D and calcium intake showed insignificant changes of 0.73 (0.55–2.50) μg and minus 18±11 mg respectively, whilst BMI and waist circumference presented respective changes of minus 0.1 ± 1.1kg/m^2^ and 1.5 (0–0.8) cm. The participants also reported no outward changes in lifestyle activities related to sun exposure during the 16 weeks intervention. It is inferred for this study population that the background characteristics and related factors of vitamin D status were unlikely to have brought about the increases in the plasma 25(OH)D concentrations shown at post-supplementation. Conversely, the increased plasma 25(OH)D concentrations shown could be attributable to primarily vitamin D3 supplementation.

**Table 3:** No significant change in key covariates of vitamin D statusshown by supplementation groups between baseline and post-supplementation (N = 106).

	Placebo 500mgCa n = 26	600 IU + 500 mg Ca n = 28	1200 IU + 500 mg Ca n = 26	4000 IU + 500 mg Ca n = 26	All participants n = 106	Adjusted ANCOVA comparison: 0 vs 600IU, p	Adjusted ANCOVA comparison: 0 vs 1200IU, p	Adjusted ANCOVA comparison: 0 vs 4000IU, p
**Dietary vitamin D intake**								
IU/day^§^	215 (146–265)	192 (166–232)	322 (250–424)	494 (391–595)	265 (190–456)	0.450	0.240	0.450
mcg/day	5.38 (3.40–6.62)	4.80 (4.15–5.80)	8.06 (6.25–10.6)	12.35 (9.78–14.88)	6.63 (4.75–11.4)			
Change (mcg/day)^§^	-0.65 (0.7–2.38)	-0.7 (0.15–1.5)	2.06 (0.5–2.35)	5.65 (3.58–5.18)	0.73 (0.55–2.50)			
**Dietary calcium intake**								
(mg/day)	333±158	365±120	387±213	367±120	376±181	0.918	0.705	0.352
Change (mg/day)	-34±54	-27±38	-9±39	2±7	-18±11			
BMI (kg/m)^2^^§^								
	21.6 (19.7–24.4)	23.0 (19.9–25.8)	21.7 (20.1–26.4)	22.0 (20.3–25.7)	21.9 (20.1–25.4)	0.485	0.835	0.795
Change (kg/m)	-0.5 ± 1.3	-0.1 ± 0.9	-0.2 ± 1.2	0.3 ± 1.0	-0.1 ± 1.1			
**Waist circumference**								
(cm)	69.2 (65.6–77.9)	72.2 (65.7–76.8)	69.7 (66.4–80.4)	69.7 (64.0–77.5)	70.4 (65.7–77.9)	0.816	0.987	0.989
Change (cm)	-3.4 (0.5–0.7)	0.6 (0.1–0.9)	-1.9 (0.1–0.9)	-0.2 (1.7–2.8)	1.5 (0–0.8)			
**Sun exposure**								
(hours /week)	3.0 (1.8–7.7)	3.9 (1.5–5.9)	3.2 (1.8–8.1)	6.4 (2.4–9.8)	3.9 (1.9–8.1)	1.000	0.904	0.685
Change (hours /week)	0	0	0	0	0			

Continuous data were expressed as mean ± SD

^§^. Skewed data were expressed as median (Q1-Q3)

Bivariate testing was done using Tukey’s HSD, and all significant values (p<0.05) were in boldface

*All significant values (p<0.05) from baseline.

## Discussion

Internationally, there is a lack of agreement on the definition of vitamin D deficiency, but there is general consensus that serum 25(OH)D levels below 30 nmol/L are deficient at all ages [35, 36]. A threshold value of 50 nmol/L has served as a guideline for vitamin D insufficiency world-wide, and since 2011 several countries have adopted the recommendations for vitamin D dietary intake levels of United States needed to achieve the plasma 25(OH)D cut-off threshold value of 50 nmol/L [37, 38].

At the baseline of this study, the majority of the participants (90.6%) plasma 25(OH)D levels that commensurate with vitamin D insufficiency (< 50nmol/L). After 16 weeks of supplementation intervention, the overall prevalence of plasma 25(OH)D insufficiency persists at a high level of 72.7%. These findings are not unexpected as previous cross-sectional studies had also reported the presence of high prevalence of vitamin D insufficiency in Malaysian female adults from urban areas. In a study of 380 subjects with a mean age of 48.5 years, approximately 87% of the females had insufficiency levels (< 50 nmol/L) of 25(OH)D [39]. Among 858 multi-ethnic adults, Shafinaz and Moy [40] documented an overall prevalence of 67.4% with vitamin D insufficiency.

As regards to change in plasma iPTH concentration, none of the 25(OH)D supplementation doses tested in this study managed to bring about a suppression of the iPTH concentration after 16 weeks of intervention. Overall, the mean iPTH concentration was shown to decrease only marginally from 4.5 ± 2.1 pmol/L at baselineto 4.3 ± 2.5 pmol/L at post-supplementation. This result is in contrast to several reports of an inverse association between serum 25(OH)D and PTH concentrations. In a review of mainly European and North American studies, Binkley, Ramamurthy, Krueger [41] suggested that circulating 25(OH)D concentrations above 75–80 nmol/L are needed for the suppression of PTH levels. Inverse relationships between serum vitamin D and PTH levels also have been reported in Asian populations at apparently lower concentrations of 25(OH)D than those reported for western populations [12, 17, 34]. As for Malaysian women of childbearing age, previous studies reported that plasma 25(OH)D threshold levels ranging from 50 to 80 nmol/L were shown to suppress PTH concentrations [21, 42]. In this intervention, the 4000 IU/day dose came closest to bringing about the plasma 25(OH)D level to 50 nmol/L at post-supplementation (49.7±26.5 nmol/L), but it did not lead to any significant iPTH suppression.

The foremost explanation for the lack of iPTH suppression in this study is postulated to be due to the presence of the high prevalence of 25(OH)D insufficiency among the subjects, even after 16 weeks of vitamin D3 supplementation. Prolonged hypovitaminosis (25(OH)D < 50 nmol/L) is known to trigger a compensatory increase in PTH with its undesirable acceleration in bone resorption, alongside other recognized adverse skeletal and non-skeletal consequences [40, 43]. Another plausible explanation for the lack of iPTH suppression in this study could be due to the supplementation duration of 16 weeks (or ~112 days), being not adequately long enough to bring about a discernible suppression of the iPTH concentration. Ramasamy [44] cited studies that showed dosing with vitamin D3 required 150–180 days to approach a steady state. Yeow et al. [45] reported changes in mean plasma 25(OH)D from <50 nmol/L to > 50 nmol/L after 24 weeks supplementation with 4000 IU/day. Another likely factor that could have restrained the suppression of iPTH may be attributable to inadequate intake of calcium among the participants. Their total calcium intake during the study period (averaging 300 mg daily from diet plus 500 mg/day from the supplement) did not meet the daily dietary recommended level of 1000 mg [18]. When blood calcium level is low or decreases, the parathyroid gland increases the secretion of PTH until calcium homeostasis is attained. A higher calcium supplementation could be considered for future interventions.

There are limitations of this study that should be considered when interpreting the results. Consistent with supplementation studies, participant compliance in this intervention was found challenging. Some studies reported that larger but less frequent dosage of vitamin D may be advantageous from a compliance perspective compared to daily doses [42]. The results reported here are based on the “intention to treat” (ITT) principle, which was favoured over the “per-protocol” procedure, for arguably preserving the prognostic balance afforded by randomisation. A study with an adequately powered sample size should be conducted to verify this study’s primary result of a lack of dose effect on PTH suppression by vitamin D3 supplementation. The findings of this study should not be generalized to the Malaysia population at large as the study subjects were purposively selected from two urban university campuses. Limitations of this study included self-reporting of dietary intake and sun exposure.

## Conclusion

In this randomised placebo-control study, vitamin D3 supplementation at 600 IU/day, 1200 IU/day or 4000 IU/day for 16 weeks did not bring about a discernible suppression of plasma intact parathyroid hormone concentration among Malaysian women of reproducible age. Plausible explanations for the lack of iPTH suppression include the presence of a high prevalence of low plasma 25(OH)D among the participants, intervention period not long enough, inadequate dietary intake of vitamin D and calcium. As protracted vitamin D insufficiency and hypocalcaemia could lead to a compensatory rise in PTH resulting in enhanced bone loss, as well as posing increasing risks of non-skeletal morbidities, further clinical trials with an adequately powered sample size should be undertaken over an appropriate study duration to verify the results obtained in this study.

## Supporting information

S1 Checklist CONSORT 2010 checklist of information to include when reporting a randomised trial*. (DOC)

S1 File (PDF)

S1 Data (XLSX)

## References

[1] Prentice A. Nutritional rickets around the world. J Steroid Biochem Mol Biol. 2013; 136:201–206. doi: 10.1016/j.jsbmb.2012.11.018 Epub 2012 Dec 7. . 23220549

[2] Rejnmark L, Bislev LS, Cashman KD, Eiríksdottir G, Gaksch M, Grübler M, et al. Non-skeletal health effects of vitamin D supplementation: A systematic review on findings from meta-analyses summarizing trial data. PLoS One. 2017;12(7):e0180512. doi: 10.1371/journal.pone.0180512 ; PMCID: PMC5501555. 28686645 PMC5501555

[3] Dibaba DT. Effect of vitamin D supplementation on serum lipid profiles: a systematic review and meta-analysis. Nutr Rev. 2019;77(12):890–902. doi: 10.1093/nutrit/nuz037 . 31407792

[4] Grant WB and Boucher BJ. Health Outcomes With Vitamin D Supplementation. JAMA. 2020;323(16):1618–1619. doi: 10.1001/jama.2020.2642 32343324

[5] Pérez-López FR, Chedraui P, Pilz S. Vitamin D supplementation after the menopause. Therapeutic Advances in Endocrinology and Metabolism.2020. doi: 10.1177/2042018820931291 ; PMCID: PMC7278294. 32551035 PMC7278294

[6] Dawson-Hughes B, Harris SS, Dallal GE. Plasma calcidiol, season, and serum parathyroid hormone concentrations in healthy elderly men and women. Am J Clin Nutr. 1997 Jan;65(1):67–71. doi: 10.1093/ajcn/65.1.67 . 8988915

[7] Bischoff-Ferrari HA, Giovannucci E, Willett WC, Dietrich T, Dawson-Hughes B. Estimation of optimal serum concentrations of 25-hydroxyvitamin D for multiple health outcomes. Am J Clin Nutr. 2006;84(1):18–28. doi: 10.1093/ajcn/84.1.18 . 16825677

[8] Roth DE, Abrams SA, Aloia J, Bergeron G, Bourassa MW, Brown KH, et al. Global prevalence and disease burden of vitamin D deficiency: a roadmap for action in low- and middle-income countries. Ann N Y Acad Sci. 2018;1430(1):44–79. doi: 10.1111/nyas.13968 Epub 2018 Sep 18. ; PMCID: PMC7309365. 30225965 PMC7309365

[9] Durazo-Arvizu RA, Dawson-Hughes B, Sempos CT, Yetley EA, Looker AC, Cao G, et al. Three-phase model harmonizes estimates of the maximal suppression of parathyroid hormone by 25-hydroxyvitamin D in persons 65 years of age and older. J Nutr. 2010;140(3):595–9. doi: 10.3945/jn.109.116681 Epub 2010 Jan 20. ; PMCID: PMC2821888. 20089790 PMC2821888

[10] Aloia JF, Talwar AS, Pollack S, Feuerman M, Yeh JK. Optimal vitamin D status and serum parathyroid hormone concentrations in African American women. The American Journal of Clinical Nutrition. 2006;84 (3) 602–609. doi: 10.1093/ajcn/84.3.602 ; PMCID: PMC2777656. 16960175 PMC2777656

[11] Lips P, Duong T, Oleksik A, Black D, Cummings S, Cox D, et al. A global study of vitamin D status and parathyroid function in postmenopausal women with osteoporosis: baseline data from the multiple outcomes of raloxifene evaluation clinical trial. J Clin Endocrinol Metab. 2001;86(3):1212–21. doi: 10.1210/jcem.86.3.7327 . 11238511

[12] Shen M, Li Z, Lv D, Yang G, Wu R, Pan J, et al. Seasonal variation and correlation analysis of vitamin D and parathyroid hormone in Hangzhou, Southeast China. J Cell Mol Med. 2020;24(13):7370–7377. doi: 10.1111/jcmm.15330 Epub 2020 May 16. ; PMCID: PMC7339220. 32415728 PMC7339220

[13] Olmos JM, Hernández JL, García-Velasco P, Martínez J, Llorca J, González-Macías J. Serum 25-hydroxyvitamin D, parathyroid hormone, calcium intake, and bone mineral density in Spanish adults. Osteoporos Int. 2016;27(1):105–13. doi: 10.1007/s00198-015-3219-6 Epub 2015 Jul 2. . 26134682

[14] Woo J, Lam CW, Leung J, Lau WY, Lau E, Ling X, et al. Very high rates of vitamin D insufficiency in women of child-bearing age living in Beijing and Hong Kong. Br J Nutr. 2008;99(6):1330–4. doi: 10.1017/S0007114507844382 Epub 2009 Oct 2. ; PMCID: PMC2790094. 17961293

[15] Ho-Pham LT, Nguyen ND, Lai TQ, Eisman JA, Nguyen TV. Vitamin D status and parathyroid hormone in a urban population in Vietnam. Osteoporos Int. 2011;22(1):241–8. doi: 10.1007/s00198-010-1207-4 Epub 2010 Apr 23. 20414642

[16] Nimitphong H, Holick MF. Vitamin D status and sun exposure in southeast Asia. Dermatoendocrinol. 2013;5(1):34–7. doi: 10.4161/derm.24054 ; PMCID: PMC3897596. 24494040 PMC3897596

[17] Yu S, Fang H, Han J, Cheng X, Xia L, Li S, et al. The high prevalence of hypovitaminosis D in China: a multicenter vitamin D status survey. Medicine (Baltimore). 2015;94(8):e585. doi: 10.1097/MD.0000000000000585 Erratum in: Medicine (Baltimore). 2015 Mar;94(11):1. PMCID: PMC4554140. 25715263 PMC4554140

[18] National Coordinating Committee on Food and Nutrition (NCCFN) (2017). Recommended Nutrient Intakes for Malaysia. A Report of The Technical Working Group on Nutritional Guidelines. Ministry of Health Malaysia, Putrajaya. Available from: https://nutrition.moh.gov.my/wp-content/uploads/2017/05/FA-Buku-RNI.pdf

[19] Institute of Medicine. Dietary reference intakes for calcium and vitamin D. Washington (DC): The National Academies Press; 2011.Available from: https://pubmed.ncbi.nlm.nih.gov/21796828/21796828

[20] Pon LW, Noor-Aini MY, Ong FB, Adeeb N, Seri SS, Shamsuddin K, et al. Diet, nutritional knowledge and health status of urban middle-aged Malaysian women. Asia Pac J Clin Nutr. 2006;15(3):388–99.https://pubmed.ncbi.nlm.nih.gov/16837432/ . 16837432

[21] Green TJ, Skeaff CM, Rockell JE, Venn BJ, Lambert A, Todd J, et al. Vitamin D status and its association with parathyroid hormone concentrations in women of child-bearing age living in Jakarta and Kuala Lumpur. Eur J Clin Nutr. 2008;62(3):373–378. doi: 10.1038/sj.ejcn.1602696 Epub 2007 Mar 7. . 17342165

[22] Hawa M, Sakinah H and Hermizi H. Calcium and Vitamin D status of Kelantanese Malay women from low income family: A population-based study. Journal of Aging Research & Clinical Practice. 2013;2(2)191–196.https://www.researchgate.net/publication/237077622_CALCIUM_AND_VITAMIN_D_STATUS_OF_KELANTANESE_MALAY_WOMEN_FROM_LOW_INCOME_FAMILY_A_POPULATION-BASED_STUDY/link/0c96051b69cfa8b7ad000000/download

[23] Pilz S, Zittermann A, Trummer C, Theiler-Schwetz V, Lerchbaum E, Keppel MH, et al. Vitamin D testing and treatment: a narrative review of current evidence. Endocr Connect. 2019;8(2): R27–R43. doi: 10.1530/EC-18-0432 ; PMCID: PMC6365669. 30650061 PMC6365669

[24] World Health Organization (1998). Obesity: Preventing and Managing the Global Epidemic. Report of a WHO Consultation on Obesity. Geneva. 11234459

[25] Tee ES, Noor MI, Azudin MN & Idris K (1997). Nutrient composition of Malaysian foods. Institute for Medical Research, Kuala Lumpur. Available from http://www.nutriscene.org.my/books/Tee%20et%20al%201997%20-%20Nutr%20Comp%20of%20Malaysian%20Foods.pdf

[26] Health Promotion Board. Dietary Guidelines 2003 for Adult Singaporeans (18–65 Years); Health Promotion Board: Singapore, 2003.Available from: https://www.nlb.gov.sg/biblio/13167301

[27] Zaleha MI, Bukhary NIB, Noriklil, Khor GL, Zaleha AM, Haslinda H, et al. Development and Validation of a Food Frequency Questionnaire for Vitamin D intake among Urban Pregnant Women in Malaysia. Malaysian Journal of Nutrition. 2015; 21: 179–190.https://www.semanticscholar.org/paper/Development-and-validation-of-a-food-frequency-for-Zaleha-Khadijah/58409d7423793c5e472a8d89233bbca1ca5a9a69

[28] Nurbazlin M, Chee WS, Rokiah P, Tan AT, Chew YY, Nusaibah AR, et al. Effects of sun exposure on 25(OH) vitamin D concentration in urban and rural women in Malaysia. Asia Pac J Clin Nutr. 2013;22(3):391–9. doi: 10.6133/apjcn.2013.22.3.15 . 23945409

[29] Institute of Medicine. Dietary Reference Intakes for Calcium and Vitamin D. Washington, D.C.: National Academies Press, 2010.https://www.ncbi.nlm.nih.gov/books/NBK56070/ doi: 10.17226/13050 21796828

[30] Ranganathan P, Pramesh CS, Aggarwal R. Common pitfalls in statistical analysis: Intention-to-treat versus per-protocol analysis. Perspect Clin Res. 2016 Jul-Sep;7(3):144–6. doi: 10.4103/2229-3485.184823 ; PMCID: PMC4936074. 27453832 PMC4936074

[31] Gupta SK. Intention-to-treat concept: A review. Perspect Clin Res 2011; 2:109–12 doi: 10.4103/2229-3485.83221 ; PMCID: PMC3159210. 21897887 PMC3159210

[32] McCoy CE. Understanding the Intention-to-treat Principle in Randomized Controlled Trials. West J Emerg Med. 2017 Oct;18(6):1075–1078. doi: 10.5811/westjem.2017.8.35985 ; PMCID: PMC3159210. 29085540 PMC5654877

[33] Hariton E, Locascio JJ. Randomised controlled trials—the gold standard for effectiveness research: Study design: randomised controlled trials. BJOG. 2018 Dec;125(13):1716. doi: 10.1111/1471-0528.15199 Epub 2018 Jun 19. ; PMCID: PMC6235704. 29916205 PMC6235704

[34] Li M, Lv F, Zhang Z, Deng W, Li Y, Deng Z, et al. Establishment of a normal reference value of parathyroid hormone in a large healthy Chinese population and evaluation of its relation to bone turnover and bone mineral density. Osteoporos Int. 2016;27(5):1907–16. doi: 10.1007/s00198-015-3475-5 Epub 2016 Jan 5. . 26733373

[35] Giustina A, Bouillon R, Binkley N, Sempos C, Adler RA, Bollerslev J, et al. Controversies in Vitamin D: A Statement From the Third International Conference. JBMR Plus. 2020 Nov 10;4(12):e10417. doi: 10.1002/jbm4.10417 ; PMCID: PMC7745884. 33354643 PMC7745884

[36] Whiting SJ, Calvo MS. Vitamin D: Nutrition Information Brief. Adv Nutr. 2021;12(5):2037–2039. doi: 10.1093/advances/nmab051 33942070 PMC8483960

[37] Institute of Medicine (US). Committee to review dietary reference intakes for vitamin D and calcium. In Dietary Reference Intakes for Calcium and Vitamin D. Eds Ross AC, Taylor CL, Yaktine AL. & Del Valle HB. Washington DC, USA: National Academies Press, 2011 Available from: https://www.ncbi.nlm.nih.gov/books/NBK56070/ doi: 10.17226/13050 21796828

[38] Bouillon R. Comparative analysis of nutritional guidelines for vitamin D. Nat Rev Endocrinol. 2017;13(8):466–479. doi: 10.1038/nrendo.2017.31 Epub 2017 Apr 7. . 28387318

[39] Moy FM and Bulgiba A. High prevalence of vitamin D insufficiency and its association with obesity and metabolic syndrome among Malay adults in Kuala Lumpur, Malaysia. BMC Public Health. 2011;11:735 doi: 10.1186/1471-2458-11-735 21943301 PMC3198705

[40] Shafinaz IS, Moy FM. Vitamin D level and its association with adiposity among multi-ethnic adults in Kuala Lumpur, Malaysia: a cross sectional study. BMC Public Health. 2016;16:232. doi: 10.1186/s12889-016-2924-1 ; PMCID: PMC4780132. 26951992 PMC4780132

[41] Binkley N, Ramamurthy R, Krueger D. Low vitamin D status: definition, prevalence, consequences, and correction. Endocrinol Metab Clin North Am. 2010;39(2):287–301. doi: 10.1016/j.ecl.2010.02.008 ; PMCID: PMC4315502. 20511052 PMC4315502

[42] Ramly M, Ming MF, Chinna K, Suboh S, Pendek R. Effect of Vitamin D Supplementation on Cardiometabolic Risks and Health-Related Quality of Life among Urban Premenopausal Women in a Tropical Country–A Randomized Controlled Trial. PLoS ONE. 2014; 9(10): e110476. doi: 10.1371/journal.pone.0110476 25350669 PMC4211685

[43] Yang K, Liu J, Fu S, Tang X, Ma L, Sun W, et al. Vitamin D Status and Correlation with Glucose and Lipid Metabolism in Gansu Province, China. Diabetes MetabSyndrObes. 2020; 7(13):1555–1563. doi: 10.2147/DMSO.S249049 ; PMCID: PMC7216298. 32440184 PMC7216298

[44] Ramasamy I. Vitamin D Metabolism and Guidelines for Vitamin D Supplementation. Clin Biochem Rev. 2020;41(3):103–126. doi: 10.33176/AACB-20-00006 ; PMCID: PMC7731935. 33343045 PMC7731935

[45] Yeow TP, Lim SL, Hor CP, Khir AS, Wan Mohamud WN, Pacini G. Impact of Vitamin D Replacement on Markers of Glucose Metabolism and Cardio-Metabolic Risk in Women with Former Gestational Diabetes—A Double-Blind, Randomized Controlled Trial. PLoS One. 2015;10(6):e0129017. doi: 10.1371/journal.pone.0129017 Erratum in: PLoS One. 2015;10(8):e0136003. PMCID: PMC4461258. 26057782 PMC4461258

