# Supplementary material for: Plasma parathyroid hormone response to vitamin D3 supplementation among women of reproductive age: A randomized double-blind placebo-control trial
Source: PLoS One. 2022 Nov 10;17(11):e0276506. doi: 10.1371/journal.pone.0276506 (PMC9648839; doi:10.1371/journal.pone.0276506)
Supplement: S1 File — (PDF) [file pone.0276506.s002.pdf]

VITAMIN D SUPPLEMENTATION AND PARATHYROID HORMONE (PTH) RESPONSE AMONG MALAYSIAN FEMALE ADULTS: DOUBLE BLIND, RANDOMISED CLINICAL TRIAL OF EFFICACY

International Medical University

School of Health Sciences

Division of Nutrition and Dietetics

CHAPTER 1: INTRODUCTION

It is well known that vitamin D is essential for bone health, and maintaining sufficient vitamin D status throughout the lifespan help reduce the osteoporotic fracture risk (1). However, recent epidemiologic and clinical studies showed that vitamin D inadequacy may have greater action and are not only associated with bone health (1, 2). Vitamin D deficiency has been associated with diseases such as osteoporosis, muscle weakness, several types of cancer, diabetes, hypertension, and cardiovascular disease (1, 3).

Vitamin D inadequacy constitutes a largely unrecognized epidemic in many populations globally. It has been reported in children, young adults and elderly adults (4). Recent data from the third National Health and Nutrition Examination Survey (NHANES) concluded that nearly 90% of women aged more than 70 years did not meet recommended daily intake of vitamin D and were at risk of vitamin D deficiency (3, 5). Data on micronutrients status in Malaysia is derived mainly from dietary studies. Challenges of such studies included poor availability of data on content of the key micronutrients in local foods (6). There are reports indicate high levels of vitamin D insufficiency among Malaysians and the emerging importance of vitamin D in health maintenance and prevention of many diseases (1, 6, 7). There are still many areas to be conducted to address this public health concern.

Due to increasing evidence showing deleterious effects of vitamin D deficiency on maternal and infant health (44, 45), there have been calls for a review of the current recommendation of 600 IU/day for child-bearing age women. Some have reported that a minimal intake of 1000 IU/day or more is required to observe benefits in skeletal and overall health (46, 47). Daily supplementation of 300 and 600 IU vitamin D2 daily for 4 weeks significantly increased the mean serum 25(OH)D of post-menopausal participants to above the deficiency level (48).

This study was designed to evaluate the efficacy of three different vitamin D supplementation dosages, namely 600 IU, 1200 IU or 4000 IU vitamin D daily for 16 weeks, on both plasma 25(OH)D and parathyroid hormone (PTH) levels among healthy Malaysian women of child-bearing age.

CHAPTER 2: BACKGROUND / LITERATURE REVIEW

2.1. Epidemiologic evidence of vitamin D situation globally, in the region and Malaysia

Micronutrients deficiencies are common globally and serious deficiencies may lead to death (6). The criteria used to defined vitamin D insufficiency in most studies was a serum 25(OH)D level below 50nmol/L (8). Vitamin D insufficiency is especially common amongst osteoporosis patients. A systematic review by Gaugris *et al.* concluded that in post-menopausal women and especially those with osteoporosis and history of fracture showed high prevalence of inadequate 25(OH)D levels (9). A global study of vitamin D status in postmenopausal women with osteoporosis showed that 24 % had 25(OH)D levels less than 25nmol/L, with the highest prevalence reported in central and southern Europe (10). Vitamin D inadequacy is also common among nonwhite populations and populations with low dietary or supplementary vitamin D intake or low exposure to sunlight. A study of Asian adults in the United Kingdom showed that 82% had 25(OH)D levels less than 30nmol/L during the summer season; the proportion increased to 94% during the winter months (11). Even children in sunny countries such as Lebanon are facing common risk with vitamin D inadequacy (12).

Recent studies carried across different countries in South and South East Asia showed widespread prevalence of vitamin D insufficiency in both sexes and all age groups of the population (8). In South India, vitamin D deficiency is equally prevalent among different population groups (13, 14). Vitamin D levels were significantly higher in rural compared to urban subjects in a population-based study (15). Prevalence of vitamin D deficiency in post-menopausal women in Thailand, Malaysia, Japan and South Korea were 47%, 49%, 90% and 92% respectively (8).

A paper by Prof. Poh Bee Koon reported that the prevalence of vitamin D insufficiency and deficiency was 27.5% and 19.7% respectively. A total of 856 children aged 4-12 years from six regions of Malaysia were studied. It is found that the serum level of vitamin D among boys (58.65 ± 1.46nmol/L) was significantly higher compared with girls (48.95 ± 1.32nmol/L). This study as well found that urban children had lower serum vitamin D level (52.83 ± 1.23nmol/L) than the rural-living children (58.14 ± 1.84nmol/L) (16). Another study on school children in Kuala Lumpur, Malaysia by Khor GL *et al.* showed that 35.3% of the children had serum 25(OH)D less than

37.5nmol/L, which indicative of vitamin D deficiency; and another 37.1% had insufficiency vitamin D concentration (>37.5 - <50nmol/L) (6). Study by TJ Green *et al.* on child-bearing age women in Jakarta and Kuala Lumpur reported over 60% of women had a 25(OH)D level of less than 50nmol/L (1). Study on effect of sun exposure on 25(OH)D concentration in urban and rural women in Malaysia indicated rural women has significantly higher 25(OH)D level (69.5nmol/L) compared to urban women (31.9nmol/L) (17). Studies by Foong MM on Malay adults in Malaysia reported that females had significant lower mean 25(OH)D level compared to males (18, 19).

2.2. Factors influencing vitamin D status

In order to maintain adequate vitamin D nutrition in the body, most adults can rely on the natural exposure to sunlight. Malaysia, with the given geographical location near the equator suggested the possibly of having yearlong sunshine. Nonetheless, the prevalence of Vitamin D insufficiency in Malaysia was reported to be considerably high (6, 19, 20). Physical factors that decreased UV-B exposure including clothing, sunscreens, and glass shielding markedly reduce or eliminate production of vitamin D in the skin (21). Cultural practice such as wearing clothing that covers the entire body, applying sunscreen and avoiding direct sunlight may attribute to vitamin D insufficiency/deficiency.

Biological factors that obstruct cutaneous vitamin D synthesis and bioavailability include skin pigmentation, medication use, body fat content and age (4, 21, 22). Cutaneous vitamin D production can be reduced as much as 99.9% with increased skin pigmentation (22, 23). Certain drugs may adversely affect metabolism or bioavailability of vitamin D, for example anticonvulsants, corticosteroids, rifampin, and cholestyramine (10, 24). There are studies shown that body mass index (BMI) and body fat content associated inversely with serum 25(OH)D levels, and directly related to PTH levels. There are limited dietary sources of vitamin D, and obtaining sufficient amount from regular diet is often difficult for many people as the few foods that are rich in vitamin D naturally were not included. Multiple factors including dietary deficiency and decreased cutaneous synthesis due to reduced ability of skin to synthesize vitamin D contribute to vitamin D deficiency among elderly. There has been association between increasing age with lower 25(OH)D levels regardless of season (25).

2.3. Biological relationships between vitamin D, PTH, calcium

The relationship between PTH and serum calcium is bifunctional. PTH regulates the serum calcium concentration while the serum calcium concentration regulates PTH secretion. As such, the stimulation of PTH secretion in response to hypocalcemia acts to restore serum calcium concentration to normal. Vitamin D status is another determinant for basal PTH value. It is widely known that vitamin D deficiency is a cause of secondary hyperparathyroidism (26). Secondary hyperparathyroidism is considered to play significant role in the pathogenesis of age-related bone loss (27, 28). It is also well recognized that serum PTH is the principal systemic determinant of bone remodeling. Although sufficient intake of vitamin D and calcium is important, study by Laufey showed that as long as vitamin D status is secured by vitamin D supplements or sufficient sun exposure, calcium intake of more than 800mg/day may be unnecessary for calcium homeostasis (28). However, there may be other beneficial effects from high calcium intake, such as protective effects in the gut lumen against colon polyp formation (28, 29, 30). Large amount of calcium been showed to be needed to meet body requirement in the absence of active calcium transport in the gut, as in vitamin D insufficiency.

2.4. Importance of sun exposure

This is interesting to understand the relationship of sun exposure and the maintenance of healthy vitamin D level in human body. It is known that sufficient sun exposure is needed to maintain adequate vitamin D level. Literature suggested that vitamin D inadequacy could be due to certain risk factors that influences the amount of UV light reaching skin surface – for example clothing that leaves little skin exposure, heat avoidance and skin colour (1). However, prolonged exposure to sunlight has reported the inverse degradation of vitamin D precursor in the skin, which then, preventing the conversion to the active vitamin. Vitamin D inadequacy in adults leads to secondary hyperthyroidism, increased risk of bone loss and higher risk of osteoporosis. Severe vitamin D deficiency causes muscle weakness, bone pain, and may develop osteomalacia (31, 32).

There is association between obesity with vitamin D deficiency. Regardless of ingested vitamin D from diet or obtained from sun exposure, vitamin D is efficiently deposited in the large body fat stores and is not bioavailable. This could be the possible reason that obese persons are chronically vitamin D deficient (33).

Vitamin D deficiency is characterized by inadequate mineralization or demineralization of the skeleton. However, effects of vitamin D are not limited to mineral homeostasis and skeletal health alone. The presence of vitamin D receptor (VDR) in other tissues suggested the importance of vitamin D in non-skeletal biological processes (31, 32, 34). Studies revealed vitamin D insufficiency in increasing risk of some common cancers, type 1 diabetes, cardiovascular diseases and osteoporosis (31). Grant reported both men and women who received minimum exposure to sunlight are at higher risk of dying of cancer (35).

At this point of time, the establishment of sun exposure recommendation is crucial but again has been challenging due to the lack of subjective measurement for sun exposure. In research, self-reported sun exposure is commonly used, but how well does the information represents actual sun exposure is poorly understood (36, 37). Until recently, the development of sunlight exposure measurement questionnaire (SEM-Q) in Pakistan showed good correlation between sun exposures against quantified exposures from UV dosimeters. The SEM-Q is recommended as a valid tool for epidemiological studies to quantify sunlight exposure (38). To date, there is only one article in Malaysia that has reported the assessment of sun exposure among Malaysian female adults (17). With the recent establishment of SEM-Q, it now leads to the validity of SEM-Q in local context given the difference in latitude, seasons (lack of) the variance in skin pigmentation and polymorphism in vitamin D receptor. These are among the factors identified affecting vitamin D synthesis. Hence, there is this need to have a validated tool to quantify sun exposure, in order to allow the recommendation of sun exposure duration to raise vitamin D to sufficient level to be determined within the local context.

2.5. Vitamin D Supplementation

Dietary vitamin D plays important role as an essential source for individuals who are unable to go outdoors frequently. Currently, the lack of availability of database on Vitamin D content in locally available food impedes the Vitamin D research. Hence, there has been no local data on vitamin D intake among Malaysian. As a result, assessment of vitamin D intake is crucial and serves as a basis to warrant vitamin D supplementation or fortification.

Health authorities in the European Union and the U.S. have set adequate intake levels of vitamin D between 400 and 600 IU/day for adults. With sun-deprived lifestyle resulting in low vitamin D synthesis in the body is the major factor for insufficient vitamin D status. Expert panels in vitamin D research have proposed 25(OH)D blood plasma level of above 75nmol/L as the optimal level. To achieve this level, supplementation studies showed that a vitamin D intake of at least 800 – 1000 IU/day is required by adults.

As based on studies, vitamin D supplementation has been estimated to prevent vitamin D deficiency in approximately 98% of general population (39). Both vitamin D supplementation and sunlight exposure have been shown to increase serum 25(OH)D levels in elderly. To protect against bone diseases and other kinds of degenerative and autoimmune diseases, adequate intake of vitamin D are very important. The Institute of Medicine (IOM) as well as the 2011 Food and Nutrition Board revised and recommended adequate intake of vitamin D up to age 70 is 600IU/day (40). Adults aged more than 70 years required 800IU/day. The upper limit (UL) for vitamin D has been revised and updated to 4,000IU/day. This dosage is defined as the highest level of daily consumption that causes no side effects in humans when used indefinitely without medical supervision (41).

It has been suggested that amounts up to 4,000IU/day of vitamin D may be needed to maintain a healthy 25(OH)D level, an intake of 400IU/day may represent a minimum. There is no reported vitamin D toxicity from long-term exposure to sunlight and no published studies showing adverse effects from dietary intake of 10,000IU dosage. This dose had been set as the No Observed Adverse Effects Level (NOAEL). Doses of 4,000IU/day for 3 months and 50,000IU/week for 2 months have been administered without toxicity (39). The European food safety authority and the US health authority have set 4000 IU/day of vitamin D as the tolerable upper intake level for adolescents and adults.

CHAPTER 3: RESEARCH OBJECTIVES AND HYPOTHESIS

Objectives

1. To determine the efficacy of vitamin D supplementation on plasma 25-hydroxyvitamin D [25(OH)D] and intact parathyroid hormone (PTH) concentrations after daily intake of supplements containing 500 mg calcium with either 0 IU, 600 IU, 1200 IU or 4000 IU vitamin D for 16 weeks.
2. To assess the influence of key covariates, namely sun exposure, vitamin D dietary intake and body mass index status on plasma 25-hydroxyvitamin D [25(OH)D] and intact parathyroid hormone (PTH) of the study participants at baseline and at 16 weeks of supplementation.

Hypothesis

1. H_0: No significant differences in mean plasma 25-hydroxyvitamin D [25(OH)D] concentrations of participants consuming daily 500 mg calcium and vitamin D supplements at 0 IU, 600 IU, 1200 IU or 4000 IU for 16 weeks

2. H_0: No significant differences in mean plasma intact parathyroid hormone (PTH) concentrations of participants consuming daily 500 mg calcium and vitamin D supplements at 0 IU, 600 IU, 1200 IU or 4000 IU for 16 weeks

3. H_0: No significant influence of sun exposure duration, dietary intake of vitamin D and body mass index on plasma 25-hydroxyvitamin D [25(OH)D] and intact parathyroid hormone (PTH) of the study participants at baseline and at 16 weeks of supplementation

CHAPTER 4: METHODOLOGY

4.1. Trial Design

This study is a stratified control trial study. It is a double-blind design, with participants randomly assigned to receive vitamin D doses of 0 IU/day, 600 IU/day, 1200 IU/day, or 4000 IU/day. The study protocol and ethics will first require approval from the International Medical University Joint Research and Ethics Committee (IMU-JC). Consent letter will be given to participants to obtain permission to participate in the study. Reasons and purpose of the study, study protocol and privacy and confidentiality will be informed. All participation shall remain as voluntary.

4.2. Participants

The eligible participants for the study are women of 3 major ethnicity (Malay, Chinese, Indian), age ranged between 20-45 years old with healthy thyroid hormone level. Participants with chronic diseases or medications thought to affect vitamin D metabolism such as hormonal use, thiazide diuretics, prednisolone, biphosphonates, tamoxifen, phenytoin, major gastrointestinal surgery, and primary hyperparathyroidism will be exclude from this study. Pregnant and lactating women will be excluded from this study as well. Participants will be covered with insurance in this research.

4.3. Study setting

The target study population is group of academic and support staffs and universities students in Klang Valley. A total of 120 women subjects from each major race (Malay, Chinese, Indian) in Malaysia will be recruited.

4.4. Intervention

a) Socio-demographic form and anthropometry measurements

A short questionnaire enquiring socio-demographic characteristics, medical history, and usage of supplementations will be given and self-administered by the participants. This served as the first-line screening of participants to confirm that the participants are free from any medications or supplementations that are known to interfere with vitamin D or

calcium metabolism. Anthropometric measurements of weight, height and waist circumference will be measured. The weight of participants will be measured using TANITA digital scale. Participants will be asked to stand on the middle of the weighing scale with barefoot, head looking straight, arms by the side and with light clothing. Then the weight will be recorded and two measurements will be taken to obtain mean body weight. The height will be measured using SECA microtoise. The microtoise will be placed to a smooth surface wall. Then, it will be calibrate until the reading is 0 when pulled on the floor surface. Similar to measuring the weight, participants are required to stand with barefoot, feet with 'v' shape lean against the wall with the heels touching the wall, head placed in Frankfurt Plane and hand by the side. Two measurements will be taken to obtain mean height. By using the mean weight and height, BMI will be calculated to determine the BMI classification of the participants. The waist circumference will be measured using a measuring tape. With the subject in standing position, it will be measure midway between the lowest rib margin and the superior border of iliac crest.

b) To assess vitamin D intake using repeated 24-hour dietary recall

A 2-days 24-hour Diet Record (include 1 weekday, and 1 weekend) will be collected. Researcher will clarify the diet record with the participants upon collection. To allow the participants estimate on portion sizes correctly, photography tools or household measurement tools will be used during the clarification. This is to prevent any under-estimation or over-estimation of portion sizes which may affect the results of the data.

c) To assess sunlight exposure by using Sun Exposure Questionnaire

The sunlight exposure of participants will be determined using a self-administered Sun Exposure Measurement Questionnaire (SEM-Q). It is a validated questionnaire widely used in other countries. Data collected from sunlight exposure measurement questionnaire (SEM-Q) in this study will be correlated with serum vitamin D.

d) Vitamin D supplementation and blood sample

Participants will be randomly divided into groups of 4 for a 16 weeks vitamin D supplementation. One of the group acts as a controlled and the other 3 grouping will be given different amount of vitamin D supplementation of 600IU/day, 1200IU/day and 4000

IU/day respectively. All groups’ supplementation included 500mg/day of calcium. Blood collection will be conducted before supplementation as a baseline data, and after 16 weeks of supplementation. The baseline blood draw before supplementation served as the second-line screening of participants. The baseline blood draw served to provides researcher the basal serum 25(OH)D of participants and to exclude any hypo- or hyperthyroidism if detected. Blood drawing will be conducted by trained physicians or phlebotomists after an overnight fast. Blood sample of 5ml will be collected. The serum will be spin down, extracted and stored at -80˚C until being analyzed. The serum 25(OH)D, and serum PTH will be analyzed. Serum vitamin D and PTH will be assayed using Centaur Analyser.

The supplements are provided by Fiatec Bioactive Sdn Bhd, Malaysia; where the raw ingredients are imported from DSM Nutritional Products. The products are prepared and packed into vegetable capsules, and will be analyze each to contain at least 90% of the required dosage amount. Vitamin D classification is categorized based on the recommendation of IOM. Serum 25(OH)D level lower than 30nmol/L considered as vitamin D deficiency; and level between 30-50nmol/L considered as vitamin D insufficiency (40). This part of the study is to determine the amount of vitamin required to provide optimum level of PTH, which in turns serves to indicate optimum absorption of calcium.

4.5. Sample size

Calculation of sample size is done with the statistical power analysis program, G*Power 3. It covers wide statistical tests and provides improved effect size calculators (43). By obtaining the last 100 data on PTH values from local research data lab, the median for 12.5, 37.5, 62.5, 87.5th percentiles and overall standard deviation (SD) were obtained. The effect size is obtained by putting in the median and SD values into the G*Power program. Using G-power with 0.7597 (effect size), a power of 0.99 and alpha significance of 0.05, the total sample size is 48 subjects.

Hence, in consideration of the research requires follow-up of 16 weeks, with a 30% possibility of dropout rate and a various conditions of exclusion criteria, a sample size of 120 participants would be needed for invitation to the study.

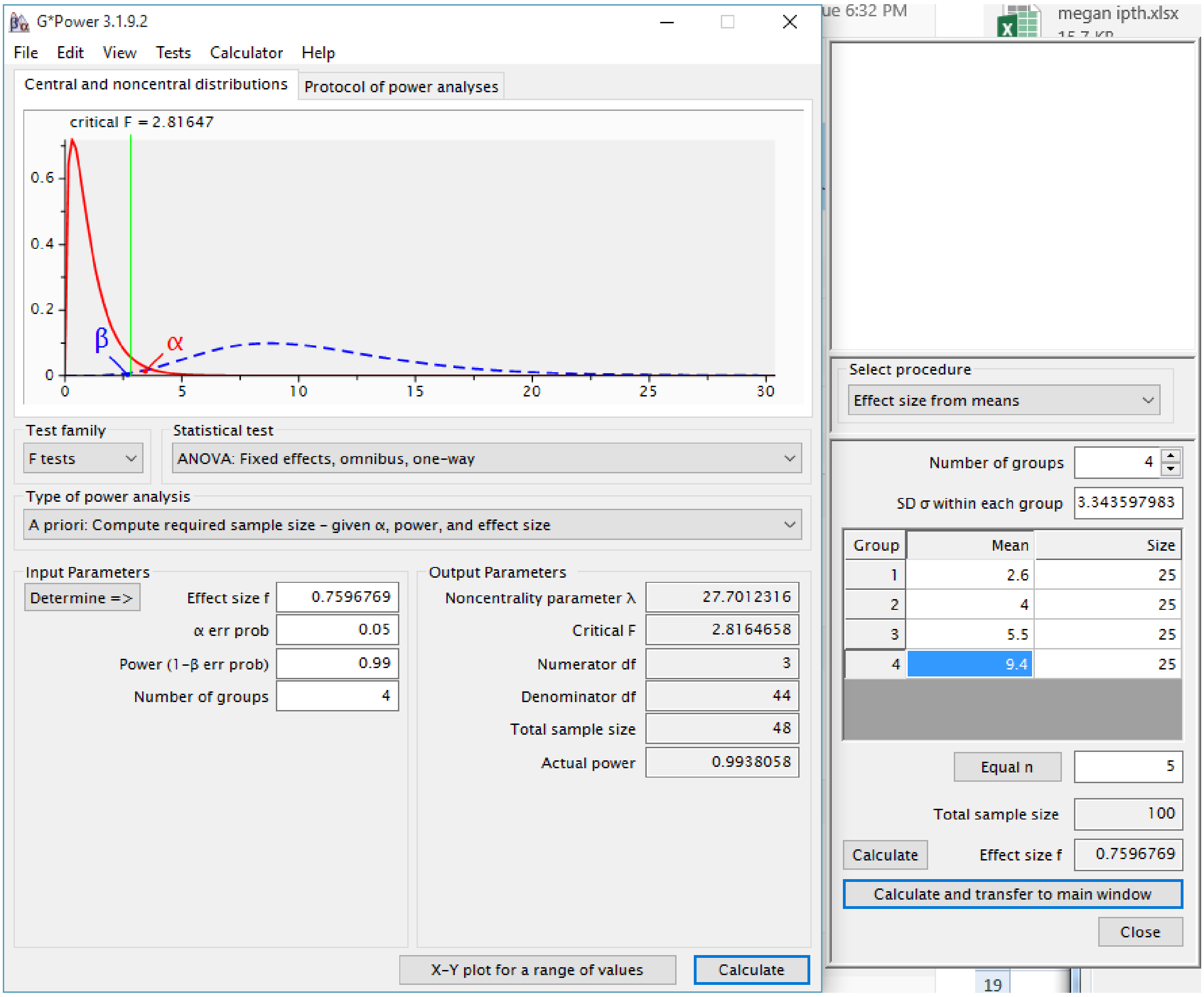

Diagram 1: Screenshot of Sample Size Calculation using G-Power

4.6. Randomisation: sequence generation

Stratified random sampling method shall be used as it allows equal distribution among each 3 ethnics as well as equal distribution for 4 supplementation groups. Each participant is masked with study code, and only the primary researcher has the master key list. Computer generated randomization of sampling into groups of four shall be used. The sample size will be divided into four groups: Placebo, 600IU/day, 1200IU/day and 4000IU/day. The divided group will then further divided equally into three ethnics group (Malays, Chinese, and Indian). The sampling method is illustrated as below:

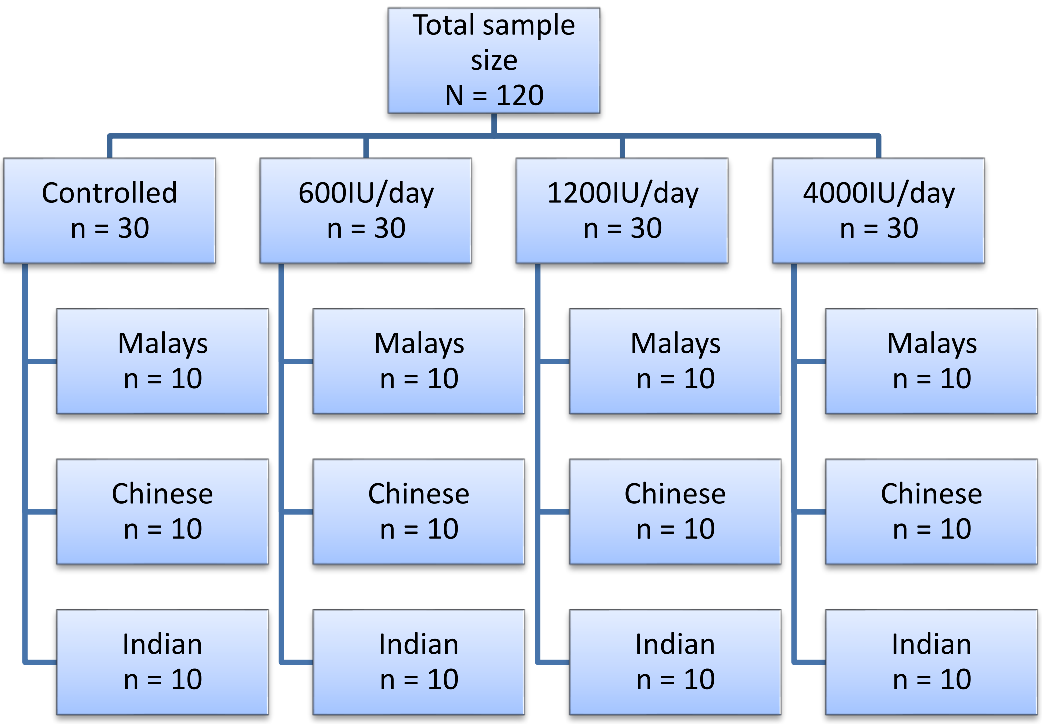

Diagram 2: Illustration of stratified sampling method

4.7. Randomisation: allocation concealment mechanism

The supplementation of vitamin D and calcium for all 4 groups are in vegetable-capsule form and identical in appearance. The supplements will be pre-packed in plastic bottles and consecutively numbered for each participant according to the randomisation schedule. Each participant is masked with a specific study code and receives the supplementation in the corresponding pre-packed bottle.

4.8. Randomisation: implementation

The researcher assigned each participant with study code. Computer generated random number list prepared by researcher. Each enrolled participants will be sequence into the random trial list. With the specific study code, the researcher will write the subjects' study code on the blood tube before passing the tubes to the phlebotomist. After blood samples collected in the blood tubes, it will be sealed into a plastic bag and send to the lab for centrifugation. The lab process will be conducted with each specific study code as identification.

4.9. Blinding

Blinding will be strictly maintained by emphasising to intervention participants that each supplement capsules adheres to healthy and safety principles. Except for interventionist (primary researcher), investigators and participants are kept blind to supplementation assignment.

4.10. Similarity of interventions

This study is similar with a previous study by MF Holick, where healthy adults aged 18-84 year old were enrolled in his study. Subjects with chronic liver and kidney disease and those taking medications, including anticonvulsants, glucocorticoids, and barbiturates, that might affect vitamin D metabolism were excluded from the study. Recruited subjects were randomly assigned in a double-blinded fashion to receive either placebo or experimental supplementation. All subjects had blood samples collected at baseline and every week for a total of 11 week. Each subject was given a dietary questionnaire at baseline to assess vitamin D and calcium consumption (42).

4.11. Statistical methods

The dietary intakes collected from the repeated 24-hour diet recall will be entered. Using the database in Nutrient Composition of Malaysian Foods (1997) and Singapore Nutrient Composition of Foods (2003), all participants' nutrients intake will be analysed. The collected data will be converted into energy and nutrients intake using Nutritionist Pro Diet Analysis Software (NutriPro Software). Further analysis of data obtained will be carried out using Statistical Package for the Social Sciences (SPSS) version 22.0. The mean, standard deviation, and/or median, inter-quartile range will be calculated to describe the demographic background of the participants and nutrients intakes from the 24-hr diet record. Data obtained will be checked for distribution pattern, whether it is normally distributed or skewed. Analysis of covariance (ANCOVA) will be used to compare between the trial groups. To determine the relationship between several independent variables and a dependant variable, multiple regressions will be used. Bland-Altman will be used to analyze the agreement of variables. The significance level is set at $p < 0.05$.

References:

[1] TJ Green, CM Skeaff, JEP Rockell, *et al.* Vitamin D status and its association with parathyroid hormone concentrations in women of child-bearing age living in Jakarta and Kuala Lumpur. Eur J Clin Nutr 2008; 62: 373-378.

[2] Michael F. Holick. Vitamin D: A Millenium Perspective. J Cell Biochem 2003; 88: 296-307.

[3] Joseph E Zerwekh. Blood biomarkers of vitamin D status. Am J Clin Nutr 2008; 87(suppl): 1087S-91S.

[4] Michael F. Holick. High Prevalence of Vitamin D Inadequacy and Implications for Health. Mayo Clin Proc. 2006; 81(3): 353-373.

[5] Moore C, Murphy MM, Keast DR, Holick MF. Vitamin D intake in the United States. J Am Diet Assoc 2004; 104: 980-3.

[6] Geok L Khor, Winnie SS Chee, Zalilah M Shariff, Bee K Poh, Mohan Arumugam, Jamalludin A Rahman, Hannah E Theobald. High prevalence of vitamin D insufficiency and its association with BMI-for-age among primary school children in Kuala Lumpur, Malaysia. BMC Public Health 2011; 11: 95.

[7] Chin KY, S Ima-Nirwana, Suraya Ibrahim *et al.* Vitamin D Status in Malaysian Men and Its Associated Factors. Nutrients 2014; 6: 5419-5433.

[8] Nidhi Malhotra and Ambrish Mithal. Vitamin D status in Asia. International Osteoporosis Foundation (IOF).

[9] Gaugris S, Heaney RP, Boonen S *et al.* Vitamin D inadequacy among post-menopausal women: a systematic review. QJM 2005; 98: 667-676.

[10] Lips P, Duong T, Oleksik A, *et al.* A global study of vitamin D status and parathyroid function in postmenopausal women with osteoporosis: baseline data from multiple outcomes of raloxifene evaluation clinical trial. J Clin Endocrinol Metab 2001; 86: 1212-1221.

[11] Pal BR, Marshall T, James C, Shaw NJ. Distribution analysis of vitamin D highlights differences in population subgroups: preliminary observations from a pilot study in UK adults. J Endocrinol 2003; 179: 119-129.

[12] El-Hajj Fuleihan G Nabulsi M, Choucair M, *et al*. Hypovitaminosis D in healthy school children. Pediatrics 2001; 107:E53.

[13] Harinarayan CV, Ramalakshmi T, Prasad UV, *et al.* High Prevalence of low dietary calcium, high phytate consumption, and vitamin D deficiency in healthy south Indians. Am J Clin Nutr 2007; 85: 1062-1067.

[14] Harinarayan CV. Prevalence of vitamin D insufficiency in postmenopausal South Indian women. Osteoporosis Int 2005; 16: 397-402.

[15] Harinarayan CV, Ramalakshmi T, Prasad UV, Sudhakar D. Vitamin D status in Andhra Pradesh: A population based study. Indian J Med Res 2008; 127: 211-218.

[16] ILSI Southeast Asia Region. Summary report: Update on Vitamin D in Human Nutrition. 12 November 2013.

[17] Musa Nurbazlin, Winnie CSS, Pendek Rokiah, *et al.* Effects of sun exposure on 25(OH) vitamin D concentration in urban and rural women in Malaysia. Asia Pac J Clin Nutr 2013; 22 (3): 391-399.

[18] Foong MM. Vitamin D status and its associated factors of free living Malay adults in a tropical country, Malaysia. J Photochem. Photobiol. B, Biol. 2011.

[19] Foong MM and A Bulgiba. High prevalence of vitamin D insufficiency and its association with obesity and metabolic syndrome among Malay adults in Kuala Lumpur, Malaysia. BMC Public Health 2011; 11: 735.

[20] Suriah A Rahman, WSS Chee, Zaitun Yassin and SP Chan. Vitamin D status among postmenopausal Malaysian women. Asia Pac J Clin Nutr 2004; 13(3): 255-260.

[21] Holick MF. McCollum Award Lecture. Vitamin D-new horizons for the 21st century. Am J Clin Nutr 1994; 60: 619-630.

[22] Kauppinen-Makelin R, Tahtela R Loyttyniemi E, Karkkainen J, Valunaki MJ. High prevalence of hypovitaminosis D in Finnish medical in- and outpatients. J Intern Med 2001; 249: 559-563.

[23] Looker AC, Dawson-Hughes B, Calvo MS, *et al.* Serum 25-hydroxyvitamin D status of adolescents and adults in two seasonal subpopulations from NHANES III. Bone 2002; 30: 771-777.

[24] Di Munno O, Mazzantini M, Delle Sedie A, *et al.* Risk factors for osteoporosis in female patients with systemic lupus erythematosus. Lupus 2004; 13:724-730.

[25] Arunabh S, Pollack S, Yeh J, Aloia JF. Body fat content and 25-hydroxyvitamin D levels in healthy women. J Clin Endocrinol Metab. 2003; 88: 157-161.

[26] Arnold J. Felsenfeld, Mariano R, and Escolastico AJ. Dynamics of parathyroid hormone secretion in health and secondary hyperparathyroidism. Clin J Am Soc Nephrol. 2007; 2: 1283-1305.

[27] Sigurdsson G, Franzson L, Steingrimsdottir L, Sigvaldason H. The association between parathyroid hormone, vitamin D and bone mineral density in 70-year-old Icelandic women. Osteoporos Int. 2000; 11: 1031-1035.

[28] Laufey S, Orvar G, Olafur S.I., Leifur F, Gunnar S. Relationship between serum parathyroid hormone levels, vitamin D sufficiency, and calcium intake. JAMA. 2005; 294: 2336-2341.

[29] Baron JA, Beach M, Mandel JS, *et al.* Calcium supplements for the prevention of colorectal adenomas. N Engl J Med. 1999; 340: 101-107.

[30] Heaney RP. Vitamin D: role in the calcium economy. In: Feldman D, Pike JW, Glorioux FH, eds. Vitamin D. Amsterdam, the Netherlands: Elsevier Inc. 2005; 773-787.

[31] Holick MF. Vitamin D: importance in the prevention of cancers, type 1 diabetes, heart disease, and osteoporosis. Am J Clin Nutr 2004; 79: 362-71.

[32] Hector F DeLuca. Overview of general physiologic features and functions of vitamin D. Am J Clin Nutr 2004; 80(suppl): 1689S-96S.

[33] Wortsman J, Matsuoka LY, Chen TC, Lu Z, Holick MF. Decreased bioavailability of vitamin D in obesity. Am J Clin Nutr 2000; 72: 690-3.

[34] Holick MF. Vitamin D: A Millenium Perspective. J Cell Biochem 2003; 88: 296-307.

[35] Grant WB. An estimate of premature cancer mortality in the U.S. due to inadequate doses of solar ultraviolet-B radiation. Cancer 2002; 94: 1867-75.

[36] Cargill, J., Lucas, R. M., Gies, P., King, K., Swaminathan, A., Allen, M. W. and Banks, E. Validation of Brief Questionnaire Measures of Sun Exposure and Skin Pigmentation Against Detailed and Objective Measures Including Vitamin D Status. Photochemistry and Photobiology 2013; 89: 219–226.

[37] Catherine A McCarty. Sunlight exposure assessment: can we accurately assess vitamin D exposure from sunlight questionnaires? 1–3 Am J Clin Nutr 2008; 87(suppl): 1097S–101S.

[38] Quratulain Humayun, Romaina Iqbal, Iqbal Azam, Aysha Habib Khan, Amna Rehana Siddiqui and Naila Baig-Ansari. Development and validation of sunlight exposure measurement questionnaire (SEM-Q) for use in adult population residing in Pakistan. BMC Public Health 2012; 12: 421.

[39] MF Holick. High prevalence of vitamin D inadequacy and implications for health. Mayo Clin Proc. March 2006; 81(3): 353-373.

[40] Institute of Medicine. Dietary Reference Intakes for Calcium and Vitamin D. Institute of Medicine: Washington, DC, USA, 2011. Reference from: https://iom.nationalacademies.org/~/media/Files/Report%20Files/2010/Dietary-Reference-Intakes-for-Calcium-and-Vitamin-D/Vitamin%20D%20and%20Calcium%202010%20Report%20Brief.pdf

[41] John Cannell. What is the upper limit and NOAEL and are they justified? Vitamin D council. [Posted: 26 Feb 2013] [Cited: 25 Aug 2015] Reference from:

https://www.vitamindcouncil.org/blog/what-is-the-upper-limit-and-noael-and-are-they-justified/#

[42] MF Holick, RM Ciancuzzo, TC Chen, *et al.* Vitamin D2 is as effective as vitamin D3 in maintaining circulating concentrations of 25-hydroxyvitamin D. J Clin Endocrinol Metab. 2008; 93: 677-681.

[43] Department of Psychology. Educational Software G-Power Version 3.0.10 from http://www.psycho.uni-duesseldorf.de/abteilungen/aap/gpower3/who-we-are, Released November 6, 2008. (Assessed September 28, 2015).

[44] Mulligan ML, Felton SK, Riek AE, Bernal-Mizrachi C. Implications of vitamin D deficiency in pregnancy and lactation. Am J Obs Gynecol. 2010;202(5):429–30.

[45] Barrett H, Mcelduff A, Au (A, Mcelduff). Vitamin D and pregnancy: An old problem revisited. Best Pract Res Clin Endocrinol Metab. 2010;24:527–39.

[46] Ross AC, Taylor CL, Yaktine AL, Valle HB Del. Dietary Reference Intakes for Calcium and Vitamin D. Institute of Medicine (IOM). Washington; 2011.

[47] NCCFN. Vitamin D. In: Noor MI, Bee Koon P, E Siong T, Geok Lin K, Shir Ley G, Hashim Z, editors. RECOMMENDED NUTRIENT INTAKES for MALAYSIA. Malaysia: Ministry of Health Malaysia; 2017. p. 252.

[48] Khrutmuang D, Panyakhamlerd K, Chatkittisilpa S, Jaisamrarn U, Taechakraichana N. Effect of multivitamin on serum 25-hydroxy vitamin D level in postmenopausal women: A randomized, double-blind, placebo-controlled trial. Osteoporos Sarcopenia. 2016;2:89–93.
